# *QuickStats:* Age-Adjusted Death Rates* for Influenza and Pneumonia,^†^ by Urbanization Level^§^ and Sex — National Vital Statistics System, United States, 2019

**DOI:** 10.15585/mmwr.mm7012a5

**Published:** 2021-03-26

**Figure:**
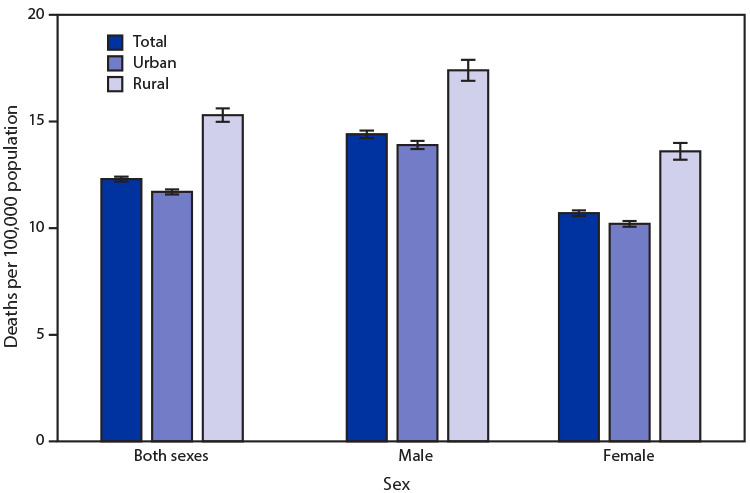
In 2019, age-adjusted death rates for influenza and pneumonia were higher among males (14.4 per 100,000) than females (10.7) and among those who lived in rural counties (15.3) compared with those who lived in urban counties (11.7). Among males, the age-adjusted death rate for influenza and pneumonia was 17.4 in rural counties and 13.9 in urban counties. Among females, the age-adjusted death rate for influenza and pneumonia was 13.6 in rural counties and 10.2 in urban counties.

**Source:** National Center for Health Statistics, National Vital Statistics System, Mortality file. https://www.cdc.gov/nchs/nvss/deaths.htm

